# Molecular docking analysis of tetracyclic triterpenoids from *Cassia fistula L.* with targets for diabetes mellitus

**DOI:** 10.6026/97320630018200

**Published:** 2022-03-31

**Authors:** Indu Sabapathy, Ireen Christopher, Vijayalakshmi Periyasamy, Rajalakshmi Manikkam

**Affiliations:** 1 DBT-BIF Centre, Holy Cross College (Autonomous), Tiruchirappalli, Tamil Nadu, India; 2 Department of Zoology, Holy Cross College (Autonomous), Tiruchirappalli, Tamil Nadu, India; 3 Department of Biotechnology, Holy Cross College (Autonomous), Tiruchirappalli, Tamil Nadu, India

## Abstract

It is of interest to develop effective drugs for diabetes mellitus. We document the molecular docking analysis data of tetra-cyclic-tri-terpenoids from *Cassia fistula L.* with targets for diabetes mellitus.

## Background:

Molecular docking is a crucial approach in computer-aided drug design that has been increasingly popular in recent years for quickly predicting the binding mechanisms and affinities of small molecules to their target molecules [1]. Tetracyclic triterpenoids are active components present in a variety of higher plants that have been studied extensively for their potential to treat diabetes and associated complications [2]. The hypoglycemic action of these tetracyclic triterpenoid compounds was previously reported in our study [3]. Therefore, it is of interest to document the molecular docking analysis data of tetracyclic triterpenoids from *Cassia fistula L.* with targets for diabetes mellitus involved in glycolysis, gluconeogenesis, glycogenolysis, de novo lipogenesis, insulin secretion and sensitivity, activation of incretin hormones, reabsorption of intestinal glucose from carbohydrate metabolism and peripheral glucose uptake (Table 1 - see PDF).

## Material and Methods:

### Preparation of receptors:

The 3D X-ray crystallographic structures of the target proteins, Glucokinase (PDB ID: 1W98), Glycogen phosphorylase (PDB ID: 1W98), Dipeptidyl peptidase (IV) (PDB ID: 1W98), Protein Tyrosine Phosphatase 1B (PDB ID: 1W98), Insulin receptor kinase (PDB ID: 1IRK), Peroxisome proliferator-activated receptor gamma (PDB ID: IZGY), α-glucosidase (PDB ID: 3WY1), Glycogen synthase kinase 3β (PDB ID: 1Q4L) were obtained from Protein Data Bank (PDB) as shown in Figure 9(see PDF). The receptors were prepared by removing the hetero-atoms and water molecules and adding polar hydrogen atoms using the Discovery Studio Visualizer 2017 R2 Client software.

### Preparation of ligands:

The structures of triterpenoid compounds were drawn using ACD/Chemsketch tool and imported in mol2 format. The 3D structures of glibenclamide and metformin were downloaded from the PubChem database in SDF format. All the ligands were transformed to PDBQT file format and saved for PyRx-Virtual screening tool.

### Molecular Docking:

The receptor proteins and ligands were docked using the PyRx Version 0.8 which enables preparing of binding site of the target protein and of screening of compound library. The results were visualized using Discovery Studio 2017 R2 Client software.

## Results and discussion:

The binding affinity scores and H bond interactions of the tetracyclic triterpenoid drugs with some known diabetes targets (Figure 9 - see PDF) were calculated and the results are shown in Table 2(see PDF). The hot spots produced by 50 percent consensus residues in all of the compounds docked in the same active site areas of the targets. The compounds' docking patterns were similar to those of the authorized diabetic medications glibenclamide and metformin. The findings revealed that the compounds had the lowest binding energy and the highest affinity for binding to receptors. The covalent contacts generated by the ligand with the active site residues of the targets are used to calculate the docking's stability (Figures 1 to 8 - see PDF).

## Conclusion:

We document the molecular docking analysis data of tetracyclic triterpenoids from *Cassia fistula L.* with targets for diabetes mellitus for further consideration.
